# Headcase Promotes Cell Survival and Niche Maintenance in the *Drosophila* Testis

**DOI:** 10.1371/journal.pone.0068026

**Published:** 2013-07-09

**Authors:** Luís Pedro F. Resende, Monica Boyle, Darrell Tran, Thomas Fellner, D. Leanne Jones

**Affiliations:** 1 Laboratory of Genetics, The Salk Institute for Biological Studies, La Jolla, California, United States of America; 2 Graduate Program in Areas of Basic and Applied Biology, Instituto Ciências Biomédicas Abel Salazar, University Porto, Porto, Portugal; 3 Molecular, Cell, and Developmental Biology, University of California-Los Angeles, Los Angeles, California, United States of America; Technische Universität Dresden, Germany

## Abstract

At the apical tip of the *Drosophila* testis, germline and somatic stem cells surround a cluster of somatic cells called the hub. Hub cells produce a self-renewal factor, Unpaired (Upd), that activates the JAK-STAT pathway in adjacent stem cells to regulate stem cell behavior. Therefore, apical hub cells are a critical component of the stem cell niche in the testis. In the course of a screen to identify factors involved in regulating hub maintenance, we identified headcase (hdc). Hub cells depleted for *hdc* undergo programmed cell death, suggesting that anti-apoptotic pathways play an important role in maintenance of the niche. Using hdc as paradigm, we describe here the first comprehensive analysis on the effects of a progressive niche reduction on the testis stem cell pool. Surprisingly, single hub cells remain capable of supporting numerous stem cells, indicating that although the size and number of niche support cells influence stem cell maintenance, the testis stem cell niche appears to be remarkably robust in the its ability to support stem cells after severe damage.

## Introduction

Adult stem cells are found in highly organized and specialized microenvironments, known as niches, within the tissues they sustain [1]. Stem cell niches are composed of a diversity of cellular and acellular components, all of them important regulators of stem cell maintenance, survival, self-renewal and the initiation of differentiation [2] [3]. Although the niche ensures the precise balance of stem and progenitor cells necessary for tissue homeostasis, stem cell niches must also be dynamic and responsive in order to modulate stem cell behavior in accordance with sudden changes in the environment, such as tissue damage, to re-establish homeostasis [4].

**Figure 1:**
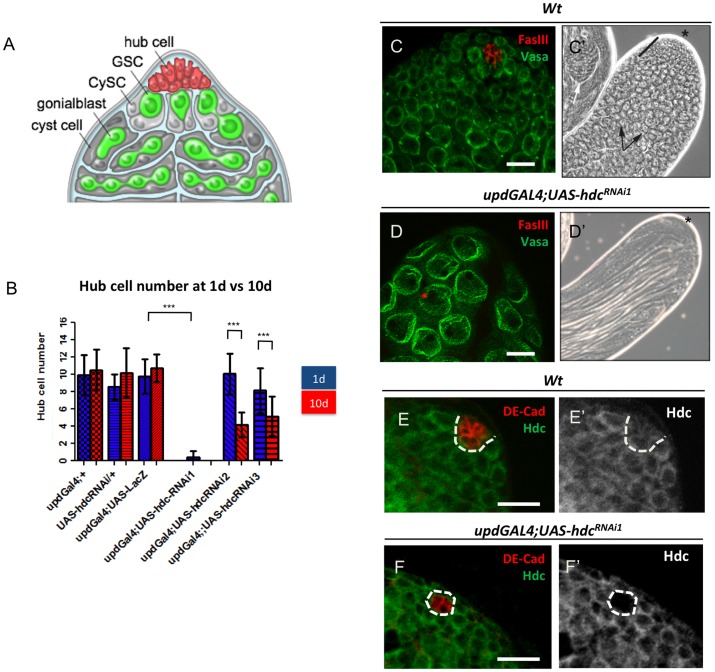
*hdc* function is required to maintain hub cells in the *Drosophila* testis. (**A**) Schematic of the male germline stem cell niche. (**B**) Hub cell quantification at 1d(blue) and 10d(red) in controls and upon reduction of *hdc*. N = 30 for each genotype/time point. Mean and SD are shown; ***P<0.001 (Kruskal–Wallis one-way analysis of variance). (**C**) Immunofluorescence image of *wild-type* (*wt*) testis. FasIII (hub, red), Vasa (germ line, green) (**C’**) Phase-contrast image of a *wt* testis. Asterisk denotes apical tip; transit-amplifying spermatogonia (black bar); spermatocytes (arrows). (**D and D’**) Reduction of *hdc* in hub cells leads to loss of hub cells and niche degeneration. Note absence of FasIII^+^ hub cells (red) and presence of large spermatocytes or mature sperm (**D’**) at the apical tip. (**E and E’**) Testis from *wt* larval (L3) male gonad showing Hdc expression in all cells at the apical tip. (**F and F’**) RNAi-mediated knock-down of *hdc* in hub cells results in loss of Hdc protein. Similar results were obtained for all RNAi lines tested. Scale bars, 20 µm. See also Table 1 and Figure 2.

The process of spermatogenesis in *Drosophila* provides a robust, genetically tractable system for analyzing the relationship between stem cells and the niche [5] [6]. Germline stem cells (GSCs) and somatic, cyst stem cells (CySCs) surround and are in direct contact with hub cells, a cluster of approximately 10 somatic cells at the tip of the testis [7] (Fig. 1A). GSCs divide to generate another GSC, as well as a daughter cell, called a gonialblast, that will undergo 4 rounds of mitosis with incomplete cytokinesis to generate a cyst of 16-interconnected spermatogonia, which will differentiate into mature sperm. CySCs also self-renew and produce cyst cells that surround and ensure differentiation of the developing spermatogonial cyst (Fig. 1A). The architecture and function of the testis stem cell niche are influenced by spatially restricted production and secretion of the JAK-STAT ligand Unpaired (Upd), exclusively by hub cells [8] [9] [10]. In addition to the JAK-STAT pathway, Hh [11] [12] [13] and BMP [14] [15] [16] [17] [18] signaling also play important roles in regulating stem cell behavior within the testis stem cell niche.

Elegant genetic studies have described pathways involved in the specification of hub cells and maturation of a functional niche during embryogenesis [19] [20] [21] [22]. However, failure to maintain the hub during development, or conditional ablation of the hub in adults leads to loss of both GSCs and CySCs (Voog *et al*, unpublished data). Similarly, aging results in changes to the apical hub, such as modest loss of cells and decreased expression of *upd* and the *Drosophila* homolog of E-cadherin, which appear to contribute to stem cell loss over time [23]. In the *Drosophila* ovary, somatic cap cells have been shown to regulate niche size and function [24]. However, in the testis, it remains unclear to what degree the overall size of the hub can influence stem cell number, how stem cells respond to damage to the niche, and how a functional niche is maintained over time. Therefore, we designed an RNAi-based screen to begin to address such questions.

## Results and Discussion

### *headcase* Function is Required for Maintenance of the Apical Hub

To identify factors involved in regulating hub size and maintenance, we sought to reduce the expression of candidate genes specifically in hub cells. We employed the bipartite GAL4-UAS system, in combination with RNAi, to reduce candidate gene expression in hub cells using the *updGAL4* ‘driver’ line. When expression of RNAi constructs was lethal or lead to developmental defects, a temperature sensitive allele of Gal80 (*Gal80^ts^*) was used to suppress Gal4 activity, and thus RNAi expression, until eclosion (hatching). Subsequently, the number of hub cells and stem cells was quantified in 1-day old (1d) and 10-day old (10d) adults.

Hub cell number varies with genetic background, even within controls; therefore, the average number of hub cells was measured in a variety of genetic backgrounds at both time points. All genotypes tested had an average hub cell number ranging between 9 and 11 (Fig. 1B; Table 1), which is consistent with previously published data for wild-type testes [7] [23]. Importantly, these results verified that hub cell number does not change significantly within 10 days post-eclosion [23].

**Table 1:** Results of candidate gene screen for regulators of hub size.

Genotype	hub cell number	+/− std dev
	1d	10d
*updGal4;+* (control)	10.06+/−2.31 (N = 31); 0%	10.94+/−2.45 (N = 31); 0%
*updGal4;UAS-LacZ* (control)	10.18+/−2.46 (N = 33); 0%	10.93+/−2.07 (N = 30); 0%
*UAS-hdcRNAi^1^/+* (control)	8.53+/−1.48 (N = 30); 0%	10.19+/−2.83 (N = 16); 0%
*updGal4;UAS-Zfh1RNAi*	11.79+/−1.88 (N = 28); 0%	11.07+/−2.40 (N = 29); 0%
*updGal4;UAS-hdcRNAi^1^*	0.43+/−0.73 (N = 30); 67%	ND
*updGal4;UAS-hdcRNAi^2^*	10.22+/−1.73 (N = 27); 0%	4.20+/−1.44 (N = 25); 0%
*updGal4;UAS-hdcRNAi^3^*	10.30+/−2.37 (N = 30); 0%	5.17+/−2.27 (N = 35); 0%
*updGal4;UAS-hdcRNAi^1^;Gal80ts* *	7.04+/−2.12 (N = 27); 0%	1.6+/−1.36 (N = 26); 35%
*updGal4;UAS-hdcRNAi^2^* ?	12.78+/−2.73 (N = 18); 0%	8.34+/−2.74 (N = 26); 0%
*updGal4;UAS-hdcRNAi^3^* ?	11.81+/−2.76 (N = 26); 0%	9.04+/−1.80 (N = 23); 0%
*updGal4;UAS-hdc^RA/C^;Gal80ts* # *	ND	9.28+/−3.21 (N = 29); 0%
*updGal4;UAS-hdc^Gsd404^;Gal80ts* # *	ND	8.79+/−2.15 (N = 28); 0%
*updGal4;;UAS-UnkRNAi*	9.69+/−1.99 (N = 16); 0%	8.71+/−2.91 (N = 17); 0%
*updGal4;UAS-CycKRNAi*	11.08+/−1.73 (N = 12); 0%	6.24+/−2.94 (N = 33); 0%
*updGal;UAS-reaper*	Embryonic lethal	Embryonic lethal
*updGal4;UAS-reaper;Gal80ts* *	8.96+/−2.40 (N = 23); 0%	9.07+/−2.08 (N = 29); 0%
*updGal4;;UAS-hid,UAS-reaper*	Embryonic lethal	Embryonic lethal
*updGal4;;UAS-hid,UAS-reaper/Gal80ts* *	9.08+/−1.56 (N = 23); 0%	5.15+/−1.49 (N = 26); 0%
*updGal4;UAS-DIAP2RNAi*	3.80+/−2.20 (N = 36); 6%	1.40+/−1.82(N = 20); 45%

Average number of hub cells (± S.D.) in testes from 1 or 10-day old males of designated genotypes. Percentage of testes with total hub cell loss also shown. See Methods for screen conditions.

* Flies raised at 18°C and shifted to 29°C upon eclosion.

?Flies raised at 18°C and shifted to 25°C upon eclosion.

# Lines used for *hdc* over-expression.

While knock-down of the majority of the candidates had no effect, reduction of the *headcase* (*hdc*) gene led to a dramatic loss of hub cells (Fig. 1B–D; Table 1). In contrast, no evident phenotype was observed when *hdc* was over-expressed in the hub (Table 1). Staining with a Hdc antibody revealed cytoplasmic expression in all cells throughout the tip of wild-type testes and demonstrated efficient knock-down of Hdc expression in hub cells upon RNAi-mediated depletion (Fig. 1E,F). Loss of hub cells upon *hdc* depletion was verified using three independent RNAi lines (see Materials and Methods).

**Figure 2:**
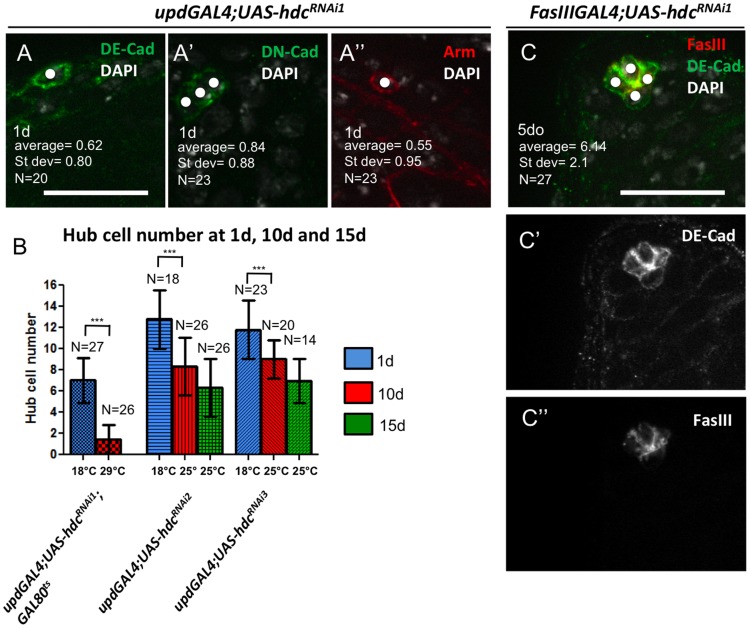
Hub cell loss is evident using multiple paradigms and is not due to developmental defects. (**A to A’”**) Strong hub cell loss marked by staining for FasIII (see Fig. 1C and F) was confirmed with other hub cell markers [DE-Cadherin (DE-Cad), DN-Cadherin (DN-cad) and Armadillo (Arm)] Hub cells pointed by white dots. (**B**) Hub cell quantification in flies where *hdcRNAi* expression by *updGal4* was suppressed at 18°C during development, and activated at 25°C (without Gal80^ts^; *hdcRNAi2 and hdcRNAi3*) or 29°C (with Gal80^ts^; *hdcRNAi1*) for 1, 10, and 15 days. Means and SD are shown; ***P<0.001 (Kruskal–Wallis one-way analysis of variance)**.** (**C**) Loss of hub cells is observed using an alternative hub driver (*FasIIIGal4*). Testis from *FasIIIGal4; UAS-hdcRNAi^1^* male at 5 days (compare to Fig. 1E); Scale bars 20 µm.

Upon depletion of *hdc* from hub cells with the strongest RNAi line tested (*hdc^RNAi1^*), the majority of testes (67%, N = 30) exhibited a complete loss of hub cells upon eclosion (Fig. 1B,D; Table 1). Remaining testes had a residual hub composed of 1 to 3 cells (Fig. 2A). To avoid interfering with hub cell specification or hub formation during development, expression of the UAS-*hdc^RNAi1^* transgene was suppressed during development using *Gal80^ts^*. No hub cell loss or niche degeneration was observed when the flies were maintained at the permissive temperature (18°C) during development (N = 35) (Fig. 2B). However, when the flies were shifted to 29°C to suppress *Gal80* activity and initiate transgene expression, complete hub loss was observed in 35% of the testes examined (N = 27) after 10 days, indicating that a reduction in hub cell number was not due to developmental defects. The remaining testes had hubs composed of 1–3 cells (Fig. 2B; Table 1).

In contrast to expression of the UAS-*hdc^RNAi1^* transgene, expression of additional *hdc* RNAi transgenes in hub cells revealed a normal number of hub cells upon eclosion, but significant loss of hub cells was observed after 10 days (Fig. 1B). Similar results were obtained when expression of these RNAi transgenes was suppressed during development and induced after eclosion (Fig. 2B; Table 1) RNAi mediated knock-down of *hdc* expression using a different hub cell-specific driver (*FasIII*Gal4) also resulted in loss of hub cells, which was confirmed by staining for multiple, well-established hub cell markers (Fig. 2A,C). Altogether, these experiments are consistent with a model in which *headcase* function is required autonomously in hub cells to prevent hub cell loss.

### Hdc Prevents Loss of Hub Cells due to Apoptosis

**Figure 3:**
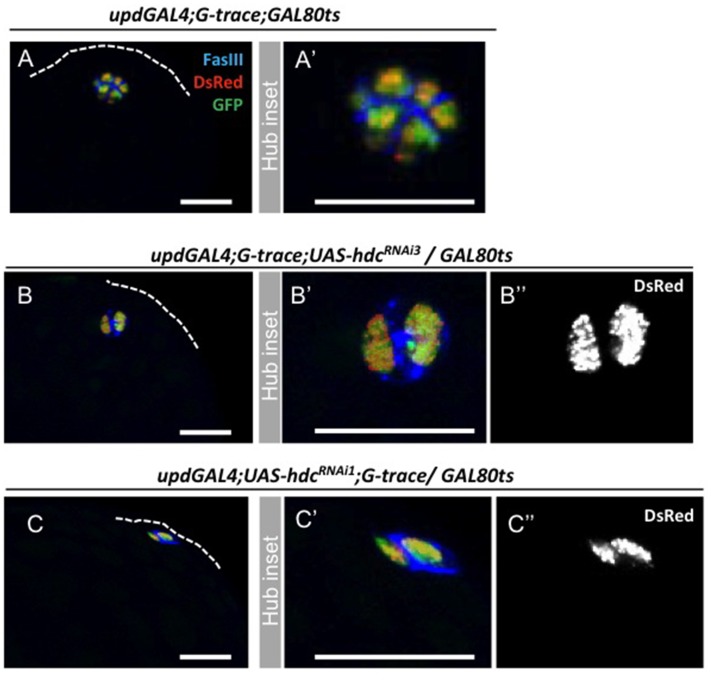
Hub cell conversion to the cyst cell lineage does not happen after *hdc* loss-of-funtion in the hub. Lineage-tracing analysis using G-TRACE in (**A, A’**) controls (genotype: *updGal4;G-TRACE;Gal80^ts^*) or (**B–C”**) upon loss of *hdc* function (genotypes: *updGal4;G-TRACE; UAS-hdcRNAi^3^/Gal80^ts^ and updGal4;UAS-hdcRNA^1;^G-TRACE/Gal80^ts^*) shows restricted expression of dsRed and GFP in hub cells. (**B” and C”**) Note no change in the levels of *upd* promoter activity (DsRed) was observed in the different categories of hub cell loss.

Several scenarios could explain the loss of hub cells in response to reduced levels of *hdc*. Cells could be lost due to programmed cell death or due to conversion to the cyst cell lineage (Voog *et al*, unpublished data). To determine the fate of lost hub cells, we first assayed for conversion to the cyst lineage by combining expression of a *hdc^RNAi^* transgene with the G-TRACE lineage-tracing cassette; G-TRACE allows both a real-time readout of GAL4 activity (dsRed), as well as a permanent lineage marker (GFP) in cells that are expressing or derived from GAL4 expressing cells (Fig. 3) [25]. There was no indication that reduced levels of *hdc* influenced the ability of hub cells to maintain their identity, as similar numbers of marked (GFP^+^) cells were observed outside the hub in testes from control (7%, N = 44), *hdcRNAi^3^* (12%, N = 85), and *hdcRNAi^1^* (15%, N = 20) G-TRACE males dissected at 5, 10 and 15 days. No significant difference was found between controls and experimental conditions (Chi-square test, P = 0.68).

**Figure 4:**
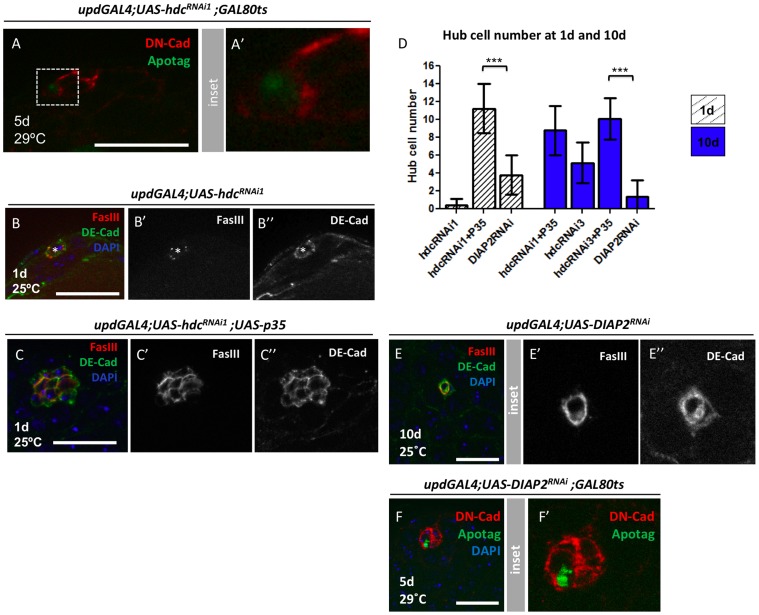
Loss of hub cells upon *hdc* reduction occurs via apoptosis. (**A**) Example of an apoptotic hub cell found after RNAi-mediated reduction in *hdc*. DN-cadherin (red), Apotag (green), Genotype: *updGal4;UAS-hdcRNAi^1^;Gal80^ts^*. (**B**) Example of a 1d *updGal4;UAS-hdcRNAi^1^* testis containing a residual hub composed of a single hub cell (*), Hub stained with both FasIII (red) and DE-cadherin (green), DAPI (DNA, blue). (**C**) Co-expression of p35 rescues the strong phenotype observed with *hdcRNAi^1^*. Compare to (B); (**D**) Hub cell number quantifications of different genotypes/time points; Mean and SD are shown; Welch’s *t*-test (***P<0.001). (**E**) Example of a compromised 10d *updGal4;UAS-DIAP2RNAi* testis; Note that DE-Cad shows two hub cells but one of them already lost FasIII staining); (**F**) Apotag positive hub cell in a *updGal4;UAS-DIAP2RNAi testis;* Scale bars 20 µm.

Next, we assayed for the presence of apoptotic hub cells upon reduction of *hdc*. Consistent with previous studies, apoptotic hub cells were rarely observed in *wild-type* testes (1/113 testes analysed) [18]. In contrast, a significant increase in the number of apoptotic hub cells was detected when *hdc* levels were reduced (12/131 testes analysed; Fisher’s exact test, P = 0.0036) (Fig. 4A). Based on hub cell counts at 1d vs 10d, approximately one hub cell is lost per day (Fig. 1B and 2B); therefore, the low frequency of testes found containing apoptotic cells is likely due to technical limitation of the detection method used. Consistent with our observations, loss of hub cells due to reduction of *hdc* was suppressed by expression of the anti-apoptotic baculovirus protein, p35, which has been shown to supress cell death efficiently in *Drosophila* (Fig. 4B–D) [26]. In addition, a loss of hub cells was observed when the pro-apoptotic factors *head involution defective* (*hid*) and *reaper* (*rpr*) were co-expressed in hub cells (Table 1). Similarly, apoptotic hub cells were detected (N = 3/39) and hub cells were lost upon RNAi-mediated knock-down of the anti-apoptotic factor, *DIAP2* (Fig. 4D–F). Based on these results, we conclude that apoptosis was a likely cause for loss of hub cells in respose to reduced levels of *hdc*. These data represent the first, direct association of this gene with programmed cell death and highlight the role of cell survival pathways in maintenance of the apical hub.

### Hub Area, Rather than Number, Determines Stem Cell Pool

**Figure 5:**
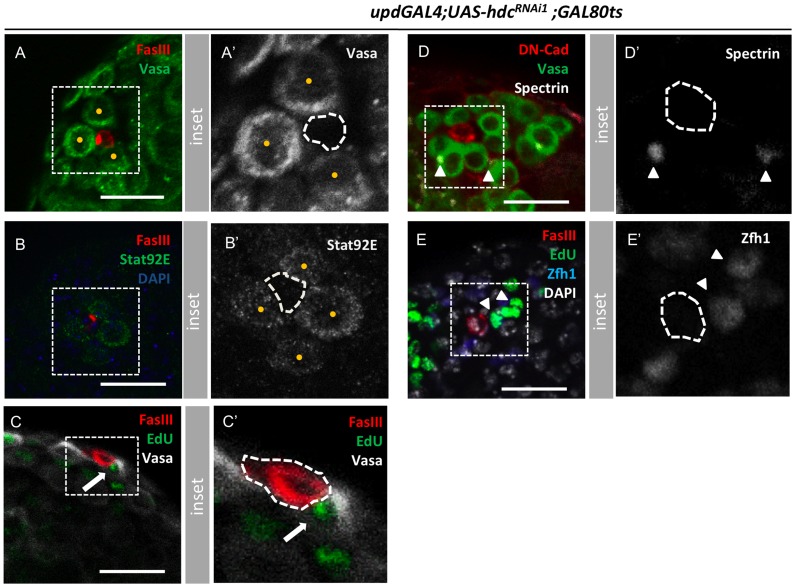
Smaller hubs are functional and capable of maintaining stem cells. (**A, A’**) Three Vasa^pos^ germ cells attached to a single cell hub (yellow dots in A’); FasIII (hub, red), Vasa (germ line, green). (**B, B’**) Four Stat92E+ GSCs (green in B, yellow dots in B’) around a 2 cell-hub. (**C, C’**) Dividing germ cell (white arrow) attached to a 2-cell hub. FasIII (hub, red), Vasa (germ line, white), EdU (green). (**D, D’**) Germ cells attached to a 1-cell hub with spherical fusomes (white arrowheads). DN-Cadherin (hub, red), Vasa (germ line, green), and α-spectrin (fusome, white). (**E, E’**) Two dividing Zfh1+ cyst cells (presumptive CySCs, white arrowheads) adjacent to a 2-cell hub. FasIII (hub, red), Zfh-1 (cyst cells, blue)**,** Edu (green), DAPI (DNA, blue). Hub is outlined in all panels by a white dashed line. In all cases, testes are from *updGal4;UAShdcRNAi^1^;Gal80^ts^* males 10 days after shifting to 29°C to induce RNAi expression. Scale bars, 20 µm. See also Video S1 and S2.

Hub cells represent a crucial component of the testis stem cell niche, serving both a structural role, as well as a localized source of self-renewal signals [8] [9] [27]. However, it is not known the degree to which the overall size of the hub influences the stem cell pool. In the course of the experiments described above, hubs consisting of single cells appeared capable of sustaining multiple GSCs, corresponding to a clear alteration in the normal hub cell: GSC ratio (Fig. 5A,B; Videos S1,S2). In addition to being adjacent to the hub, these germ cells maintained hallmarks of GSCs, including spherical spectrosomes, positive staining for Stat92E, and markers of cell cycle progression (Fig. 5B–D). Similarly, dividing Zfh1^+^ cyst cells, presumptive CySCs [16], were found adjacent to the hub (Fig. 5E), indicating that remaining hub cells are capable of supporting the maintenance of adjacent stem cells.

**Figure 6:**
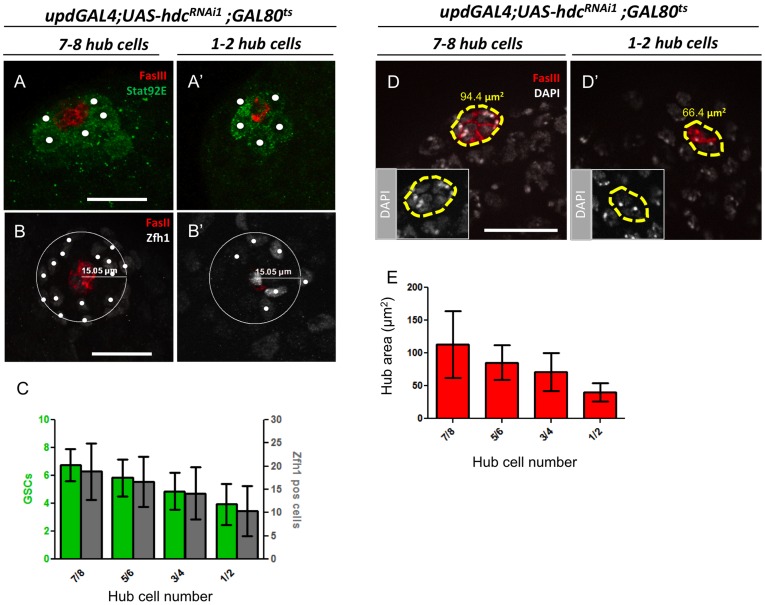
Alterations in GSCs, CySCs, and hub area during progressive hub cell loss. (**A, B and D left panels**) Testes with 7–8 hub cells (FasIII, red) from 1-day old *updGal4;UAS-hdcRNAi^1^;Gal80^ts^* males raised at 18°C. (**A’, B’ and D’, right panels**) Testes with 1–2 hub cells from *updGal4;UAS-hdcRNAi^1^;Gal80^ts^* males after 7–9 days at 29°C to induce transgene expression. (**A, A’**) GSCs were counted as Stat92E^+^ germ cells (green) contacting the hub (**B, B’**) CySCs were counted as Zfh1^+^ cells (white) within a 15 µm radius from the center of the hub. **C**) Graph representing hub cell:GSC:CySC ratio during progressive hub cell loss; N≥20 testes for each genotype/timepoint; (**D, D’**) Hub area was measured based on FasIII^+^/DAPI^+^ cells. (**E**) Graph of hub area during hub cell loss. N≥20 testes for each genotype/timepoint. Means and SD are shown. Scale bars, 20 µm.

To obtain a better understanding of how hub cell number and stem cell loss is correlated, hub cell and stem cell numbers were quantified at additional time points between 1 and 10 days. Samples were divided into classes according to hub cell number (1–2;3–4;5–6;7–8), and for each of these classes the number of GSCs and CySCS was counted. GSCs were scored as germ cells adjacent to the hub and positive for Stat92E (Fig. 6A,A’), while presumptive CySCs were scored as Zfh1^+^ cells within a 15 µm distance from the center of the hub (Fig. 6B, B’). Both GSCs and CySCs were sensitive to alterations in hub cell number, as a decrease in the number of both stem cell populations was observed as hub cells numbers drop (Fig. 6C). However, loss was not as dramatic as would have been predicted if the ratio between stem cells and niche cells was maintained. Hubs composed of only one or two cells maintained an average of approximately half of the original number of both GSCs and CySCs (Fig. 6A–C, Video S2). Indeed, total stem cell loss and loss of spermatogonia were only observed after a complete loss of the hub (N>30).

Because proximity to the hub is essential for both GSCs and CySCs to maintain stem cell identity, we hypothesized that stem cell loss might correlate more closely with reduction of hub area, rather than total hub cell number. The average hub area in previously defined categories revealed that a 5-fold reduction in hub cell number (7.5 vs 1.5 hub cells) corresponded to only a 2.8 fold reduction in the hub cell area (112.98 to 40.12 µm^2^) (Fig. 6D, E). Strong *upd* promoter activity was detected in hub cells in both controls and during progressive hub cell loss in response to reduction of *hdc* (Fig. 3B” and C”). In addition, no obvious differences were noted in the levels of JAK-STAT pathway activation in GSCs upon hub cell loss, as determined by Stat92E staining in adjacent GSCs (Fig. 6A, A’); therefore, remaining hub cells appear to be functional. This is in contrast to the loss of stem cells in older animals, which occurs due to a reduction in *upd*, and perhaps DE-cadherin, rather than a dramatic reduction in hub cell number [23].

### Conclusions

**Figure 7:**
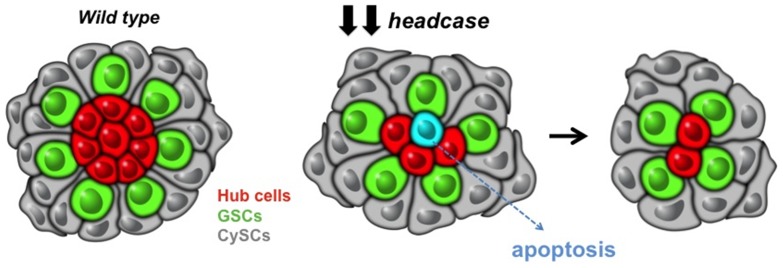
Reduced *headcase* levels in hub cells results in loss of hub cells due to apoptosis. As hub cells are lost, stem cell niche architecture changes. However, severely compromised hubs composed of only 1 or 2 cells remain functional and capable of maintaining active stem cells around them.

Although a considerable amount is known regarding the initial formation of the apical hub the testis, much less is known about how its function is regulated throughout life. Here, we identified *headcase* (*hdc*) as a novel factor involved in maintenance of hub cells. Upon depletion of *hdc*, hub cells undergo apoptosis, stem cells are lost, and the niche degenerates (Fig. 7). A molecular function for Hdc has not been described previously; however, our data are consistent with a possible role for Hdc in promoting cell survival. This conclusion is supported by the identification of *hdc* in a screen for specific regulators of photoreceptor development, where the *hdc* mutant phenotype resembled that of *crumbs,* a gene required to prevent programmed cell death in ommatidia [28] [29]. Interestingly, loss of *hdc* suppressed the overgrowth phenotype induced by ectopic activation of the JAK-STAT pathway in the *Drosophila* eye [30]. Although not tested directly, it is tempting to speculate that mutations in *hdc* suppressed overgrowth in the eye by leading cells to enter apoptosis. Therefore, a putative role in preventing apoptosis may be conserved amongst tissues. Other published studies on *headcase* have proposed a role for this gene in tracheal branching and neuronal pruning [31] [32].

Conditional ablation of hub cells through reduction of *hdc* also permitted a detailed investigation of the response of stem cells to damage to the niche and revealed how the support cell: stem cell ratio scales in the *Drosophila* testis. In essence, we find that the overall area of the apical hub appears to be more important than a specific number of hub cells. The importance of the area defined by hub cells and its effect on the stem cell pool is supported by findings in other systems, where stem cells are regulated by signals from the support cells that act in a short-range fashion. For example, in the *Drosophila* ovary, where cap cells play an analogous role to hub cells, an expansion of cap cells leads to an increase in the number of GSCs, and the area defined by cap cells appears to limit the number of GSCs [24] [33]. Additional parallels can be found in mammalian stem cell niches, such as in the small intestine. In this system, genetic ablation of Paneth cells in vivo leads to a loss of Lgr5^+^ crypt base columnar (CBC) cells (intestinal stem cells). However, similar to our observations in the *Drosophila* testis, in crypts where a single Paneth cell is remaining, multiple CBCs can be found clustered around it [34]. Altogether, our data underscore the role of cell survival pathways in maintaining niche size by promoting the survival of support cells. These results also provide insight into how stem cell number is altered as a consequence of damage to the niche, which will be important for the development and utilization of synthetic stem cell niches for the maintenance and expansion of stem cells *in vitro* for use in regenerative medicine.

## Materials and Methods

### Fly Husbandry and Stocks

Flies were raised on standard cornmeal-molasses-agar medium. Male progeny from experimental crosses were collected and maintained with less than 30 flies per vial. Flies were turned onto fresh food every two days.

The following stocks were used; more information on them can be found in Flybase (http://flybase.bio.indiana.edu): *updGal4*; *FasIIIGal4*; *UAS-p35* (Bloomington stock center #5072 and #5073), *Gal80^ts^* (Bloomington stock center #7018), *UAS-reaper and UAS-reaper,UAS-hid* (gifts from E. Rulifson*), UAS-lacZ^NLS^* (Bloomington stock center #3956*)*; *UAS-DsRed*,*UAS-flp*, *ubi>stop>GFP*/Cyo;MKRS/TM6b and Cyo/Sco; *UAS-DsRed,UAS-flp, ubi>stop>GFP*/TM6b (G-TRACE cassettes on II and III) (gift from U. Banerjee) [Evans et al, 2009]; *hdc*RNAi lines used were from Vienna *Drosophila* stock center and labeled as *UAS-hdcRNAi^1^, UAS-hdcRNAi^2^* and *UAS-hdcRNAi^3^*, *UAS-unkRNAi, UAS-cycKRNAi, and UAS-DIAP2^RNAi^* corresponding to VDRC#45069, VDRC#104322 and VDRC#39877, VDRC#4267, VDRC#110774, and #2973 respectively. Two *hdc* sequences that map across the gene are target by the three RNAi lines: UAS*hdc*RNAi^2^ (milder phenotype) targets all three isoforms while UAS*hdc*RNAi^1^ (stronger phenotype) and UAS*hdc*RNAi^3^ (milder phenotype) are the same construct but inserted in diferent chromosomes and only target two of the 3 isoforms. Note that hub cell loss could not be tested using *hdc* mutants, as all alleles tested (*hdc^50^, hdc^Fus-6^, hdc^43^, hdc^B4-3-20^* and *hdc^KG09851^*) exhibited embryonic/larval lethality.

For *hdc* over-expression two lines were used: *UAS-hdc^GSD404^* (gift from D. Van Meyel), and *UAS-hdc^RA/C^.* To generate transgenic flies carrying *UAS-hdc^RA/C^*, the full length *hdc* sequence, corresponding to isoforms A/C, was amplified via PCR and cloned into the pUAST-attB vector to allow targeted integration into the att2 site on the third chromosome. Embryo injections were performed by Genetic Services Inc (Sudbury, MA).

### Candidate RNAi Screen

Male flies carrying *RNAi* transgenes targeting candidate genes were crossed to virgin females carrying the *updGal4* driver. Crosses were set at 25°C and male flies were dissected upon eclosion (1d) or collected and maintained at 25°C before dissection at 10 days (10d).

Suppression of expression of the UAS-*rpr,* UAS-*rpr+*UAS-*hid,* UAS-*hdcRNAi^1^ and G-TRACE* constructs during development was achieved using a temperature sensitive allele of Gal80 (*Gal80^ts^)*. Flies were raised at 18°C and shifted to 29°C upon eclosion (day 1) to activate transgene expression for 10 days, or as otherwise noted. Expression of *UAS-hdcRNAi^2^* and *UAS-hdcRNAi^3^* was suppressed during development by raising flies at 18°C (ie., *Gal80^ts^* was not used).

### Antibodies

Testes were stained with: rabbit anti-Vasa (1∶2,000) (gift from P. Lasko); mouse anti-Hdc(1∶5) (gift from R. White); guinea pig anti-Zfh-1 (1∶3,000) (gift from C. Doe); rabbit anti-Stat92E (1∶800) (gift from D. Montell); mouse anti-Fasciclin III (7G10) (1∶10); rat anti-DE-cadherin (1∶20), rat anti-DN-cadherin (1∶20) and mouse anti-armadillo (1∶15) (Developmental Studies Hybridoma Bank (developed under the auspices of the National Institute of Child Health and Human Development and maintained by The University of Iowa, Department of Biological Sciences); rabbit anti-GFP(1∶5000) (Molecular Probes). Secondary antibodies were diluted 1∶500 (Molecular Probes).

### Immunofluorescence and Microscopy

Phase contrast images of squashed testes were obtained using a Leica DM5000 microscope equipped with a DC500 camera, using Firecam imaging software (version 1.7.1; Leica Microsystems). Immunofluorescence (IF) microscopy was performed on whole-mount testes dissected into PBS and fixed in 2% PFA as previously described (Boyle et al., 2007). Images were obtained using either a Zeiss LSM 710 Laser Scanning confocal microscope or Zeiss Axiovert 200 microscope equipped with Apotome. Samples were mounted in Vectashield mounting medium with DAPI (Vector Laboratories). Pictures were analyzed in AxioVision (version 4.8; Carl Zeiss) and Adobe Photoshop software (Mountain View, CA).

### Cell Counts and Hub Area Measurements

All experiments involving cell counts (hub cells, GSCs or CySCs) were performed using at least 10 sections (Z-stacks). Hub cells were counted as DAPI^+^ nuclei that were FasIII^+^, using a 63X objective. Others markers including DE-Cad, DN-Cad, and Arm were used simultaneously with FasIII in a diversity of experimental paradigms and no noteworthy discrepancy was observed. GSCs were counted as STAT92E positive germ cells contacting the hub. Presumptive CySCs were scored as Zfh1^+^ found in a 15 µm radial distance from the center of the hub. Hub area was measured using the AxioVision (version 4.8; Carl Zeiss) software to calculate the area defined by FasIII^+^ cells. Area, rather than volumetric measurements, was used due to the fact that our data (Video S1 and S2) suggested that the hub was a flat disc, rather than a sphere, although this could be a consequence of our protocol for sample preparation and mounting.

### Statistical Analysis

For the purpose of comparing hub cell averages between multiple genotypes/time points found on Figure 1 and Figure 2 the Kruskal–Wallis one-way analysis of variance test was used, followed by a Dunn’s multiple comparison test. For Figure 4, a Welch’s *t*-test was used to determine the statistical significance of the difference in hub cell averages between pairs of columns (*hdc*RNAi1 vs *hdc*RNAi1+p35) (*hdc*RNAi3 vs *hdc*RNAi3+p35). For both comparisons, p-values were represented in graphs using the following asterisk code: * P^2^0.05, **P^2^0.01, ***P^2^0.001. Statistical significance of the frequency of apoptotic hub cells was tested with the Fisher’s exact test, two tailed. A Chi-square test was used to analyze the G-trace data and the frequency of testes with hub-restricted vs non-restricted GFP expression.

### Apoptosis Assay

Testes were fixed and processed using the Chemicon ApoTag Fluorescien Kit, according to manufacturer’s instructions, followed by immunofluorescence as described above.

### Ex vivo EdU Incorporation

EdU incorporation was performed and analyzed using the Click-iT EdU Imaging Kit (Invitrogen), with the following modifications. All procedures were performed at room temperature with minimal exposure to light. Crude dissection of testes was performed in 1X Ringer’s buffer and then transferred immediately to 1X Ringer’s buffer in a glass embryo dish for no more than 10 minutes. Testes were subsequently transferred to 30 µM EdU diluted in 1X Ringer’s buffer for 30′. After incorporation, testes were fixed for 20′ in 4% paraformaldehyde diluted in 1X PBS, followed by two washes with 1X PBST (0.5% Triton-X100) and blocked with 3% BSA in 1X PBS. Testes were bathed in the Click-iT reaction cocktail for 30 minutes. IF was performed as indicated above.

## Supporting Information

Video S1 **3D reconstruction of GSCs surrounding a normal size hub and a compromised hub. S1)** Example of a testis from a 1 day-old *updGal4;UAS-hdcRNAi^1^;Gal80^ts^* male highlighting the attachment of GSCs (Stat92E^+^,green) to a hub containing 7 cells (FasIII, red). DAPI (DNA, blue). b).
(MOV)

Video S2 **3D reconstruction of GSCs surrounding a normal size hub and a compromised hub. S2)** Same genotype as S1) but 8day old and with a compromised hub containing only 1 cell.
(MOV)

## References

[1] Schofield R (1978) The relationship between the spleen colony-forming cell and the haemopoietic stem cell. *Blood Cells* 4: 7–25. 747780

[2] Morrison SJ, Spradling AC (2008) Stem cells and niches: mechanisms that promote stem cell maintenance throughout life. *Cell* 132: 598–611. 18295578 10.1016/j.cell.2008.01.038 PMC4505728

[3] Jones DL, Wagers AJ (2008) No place like home: anatomy and function of the stem cell niche. *Nat. Rev. Mol. Cell Biol.* . 9: 11–21. 10.1038/nrm2319 18097443

[4] Voog J, Jones DL (2010) Stem cells and the niche: a dynamic duo. *Cell Stem Cell* 6: 103–115. 20144784 10.1016/j.stem.2010.01.011 PMC3012646

[5] Losick VP, Morris LX, Fox DT, Spradling A (2011) Drosophila Stem Cell Niches: A Decade of Discovery Suggests a Unified View of Stem Cell Regulation. *Developmental Cell* 21: 159–171. 21763616 10.1016/j.devcel.2011.06.018 PMC6894370

[6] De Cuevas M, Matunis EL (2011) The stem cell niche: lessons from the Drosophila testis. *Development* 138: 2861–2869. 21693509 10.1242/dev.056242 PMC3119301

[7] Hardy RW, Tokuyasu KT, Lindsley DL, Garavito M (1979) The germinal proliferation center in the testis of Drosophila melanogaster. *J. Ultrastruct. Res.* . 69: 180–190. 10.1016/s0022-5320(79)90108-4 114676

[8] Tulina N, Matunis E (2001) Control of stem cell self-renewal in Drosophila spermatogenesis by JAK-STAT signaling. *Science* 294: 2546–2549. 11752575 10.1126/science.1066700

[9] Kiger AA, Jones DL, Schulz C, Rogers MB, Fuller MT (2001) Stem cell self-renewal specified by JAK-STAT activation in response to a support cell cue. *Science* 294: 2542–2545. 11752574 10.1126/science.1066707

[10] Harrison DA, McCoon PE, Binari R, Gilman M, Perrimon N (1998) Drosophila unpaired encodes a secreted protein that activates the JAK signaling pathway. *Genes Dev.* . 12: 3252–3263. 10.1101/gad.12.20.3252 PMC317220 9784499

[11] Amoyel M, Sanny J, Burel M, Bach EA (2013) Hedgehog is required for CySC self-renewal but does not contribute to the GSC niche in the Drosophila testis. *Development* . 140: 56–65. 10.1242/dev.086413 PMC3513992 23175633

[12] Michel M, Kupinski AP, Raabe I, Bökel C (2012) Hh signalling is essential for somatic stem cell maintenance in the Drosophila testis niche. *Development* 139: 2663–2669. 22745310 10.1242/dev.075242

[13] Zhang Z, Lv X, Jiang J, Zhang L, Zhao Y (2013) Dual roles of Hh signaling in the regulation of somatic stem cell self-renewal and germline stem cell maintenance in Drosophila testis. *Cell Res*. doi:10.1038/cr.2013.29. 10.1038/cr.2013.29 PMC3616438 23419515

[14] Shivdasani AA, Ingham PW (2003) Regulation of stem cell maintenance and transit amplifying cell proliferation by tgf-beta signaling in Drosophila spermatogenesis. *Curr. Biol.* . 13: 2065–2072. 10.1016/j.cub.2003.10.063 14653996

[15] Kawase E, Wong MD, Ding BC, Xie T (2004) Gbb/Bmp signaling is essential for maintaining germline stem cells and for repressing bam transcription in the Drosophila testis. *Development* 131: 1365–1375. 14973292 10.1242/dev.01025

[16] Leatherman JL, Dinardo S (2008) Zfh-1 controls somatic stem cell self-renewal in the Drosophila testis and nonautonomously influences germline stem cell self-renewal. *Cell Stem Cell* 3: 44–54. 18593558 10.1016/j.stem.2008.05.001 PMC2601693

[17] Zheng Q, Wang Y, Vargas E, DiNardo S (2011) magu is required for germline stem cell self-renewal through BMP signaling in the Drosophila testis. *Dev. Biol.* . 357: 202–210. 10.1016/j.ydbio.2011.06.022 PMC3431605 21723859

[18] Michel M, Raabe I, Kupinski AP, Pérez-Palencia R, Bökel C (2011) Local BMP receptor activation at adherens junctions in the Drosophila germline stem cell niche. *Nat Commun* 2: 415. 21811244 10.1038/ncomms1426

[19] Boyle M, DiNardo S (1995) Specification, migration and assembly of the somatic cells of the Drosophila gonad. *Development* 121: 1815–1825. 7600996 10.1242/dev.121.6.1815

[20] Le Bras S, Van Doren M (2006) Development of the male germline stem cell niche in Drosophila. *Dev. Biol.* . 294: 92–103. 10.1016/j.ydbio.2006.02.030 16566915

[21] Wawersik M, Milutinovich A, Casper AL, Matunis E, Williams B, Van Doren M (2005) Somatic control of germline sexual development is mediated by the JAK/STAT pathway. *Nature* 436: 563–567. 16049490 10.1038/nature03849 PMC1421378

[22] Sheng XR, Posenau T, Gumulak-Smith JJ, Matunis E, Van Doren M, et al (2009) Jak-STAT regulation of male germline stem cell establishment during Drosophila embryogenesis. *Dev. Biol.* . 334: 335–344. 10.1016/j.ydbio.2009.07.031 PMC2777977 19643104

[23] Boyle M, Wong C, Rocha M, Jones DL (2007) Decline in self-renewal factors contributes to aging of the stem cell niche in the Drosophila testis. *Cell Stem Cell* 1: 470–478. 18371382 10.1016/j.stem.2007.08.002

[24] Song X, Call GB, Kirilly D, Xie T (2007) Notch signaling controls germline stem cell niche formation in the Drosophila ovary. *Development* 134: 1071–1080. 17287246 10.1242/dev.003392

[25] Evans CJ, Olson JM, Ngo KT, Kim E, Lee NE, et al (2009) G-TRACE: rapid Gal4-based cell lineage analysis in Drosophila. *Nat.* . *Methods* 6: 603–605. 10.1038/nmeth.1356 PMC2754220 19633663

[26] Hay BA, Wolff T, Rubin GM (1994) Expression of baculovirus P35 prevents cell death in Drosophila. *Development* 120: 2121–2129. 7925015 10.1242/dev.120.8.2121

[27] Resende LPF, Jones DL (2012) Local signaling within stem cell niches: insights from Drosophila. *Curr. Opin. Cell Biol.* . 24: 225–231. 10.1016/j.ceb.2012.01.004 PMC4821423 22296770

[28] Gambis A, Dourlen P, Steller H, Mollereau B (2011) Two-color in vivo imaging of photoreceptor apoptosis and development in Drosophila. *Dev. Biol.* . 351: 128–134. 10.1016/j.ydbio.2010.12.040 PMC3051417 21215264

[29] Johnson K, Grawe F, Grzeschik N, Knust E (2002) Drosophila crumbs is required to inhibit light-induced photoreceptor degeneration. *Curr. Biol.* . 12: 1675–1680. 10.1016/s0960-9822(02)01180-6 12361571

[30] Bach EA, Vincent S, Zeidler MP, Perrimon N (2003) A sensitized genetic screen to identify novel regulators and components of the Drosophila Janus kinase/Signal transducer and activator of transcription pathway. *Genetics* 165: 1149–1166. 14668372 10.1093/genetics/165.3.1149 PMC1462825

[31] Loncle N, Williams DW (2012) An interaction screen identifies headcase as a regulator of large-scale pruning. *J. Neurosci.* . 32: 17086–17096. 10.1523/JNEUROSCI.1391-12.2012 PMC3541709 23197702

[32] Steneberg P, Englund C, Kronhamn J, Weaver TA, Samakovlis C (1998) Translational readthrough in the hdc mRNA generates a novel branching inhibitor in the Drosophila trachea. Genes Dev. 1 12(7): 956–67. 10.1101/gad.12.7.956 PMC316679 9531534

[33] Xie T, Spradling AC (2000) A niche maintaining germ line stem cells in the Drosophila ovary. *Science* 290: 328–330. 11030649 10.1126/science.290.5490.328

[34] Sato T, Van Es JH, Snippert HJ, Stange DE, Vries RG, et al (2011) Paneth cells constitute the niche for Lgr5 stem cells in intestinal crypts. *Nature* 469: 415–418. 21113151 10.1038/nature09637 PMC3547360

